# Fluorescence in situ hybridization (FISH) as an irreplaceable diagnostic tool for Williams-Beuren syndrome in developing countries: a literature review

**DOI:** 10.1590/1984-0462/2023/41/2022125

**Published:** 2023-07-10

**Authors:** Bianca Soares Carlotto, Desirée Deconte, Bruna Lixinski Diniz, Priscila Ramires da Silva, Paulo Ricardo Gazzola Zen, André Anjos da Silva

**Affiliations:** a Universidade Federal de Ciências da Saúde de Porto Alegre, Porto Alegre, RS, Brazil.; b Irmandade da Santa Casa de Misericórdia de Porto Alegre, Porto Alegre, RS, Brazil.; c Universidade do Vale do Rio dos Sinos, São Leopoldo, RS, Brazil.

**Keywords:** Williams syndrome, Williams-Beuren syndrome, Fluorescence in situ hybridization, Literature review, Síndrome de Williams, Síndrome de Williams-Beuren, Hibridização in situ fluorescente, Revisão de literatura

## Abstract

**Objective:**

The aim of this study was to sum up and characterize all Williams-Beuren syndrome cases diagnosed by fluorescence in situ hybridization (FISH) since its implementation, as well as to discuss FISH as a cost-effective methodology in developing countries.

**Data source:**

From January 1986 to January 2022, articles were selected using the databases in PubMed (Medline) and SciELO. The following terms were used: Williams syndrome and In Situ Hybridization, Fluorescence. Inclusion criteria included Williams-Beuren syndrome cases diagnosed by FISH with a stratified phenotype of each patient. Only studies written in English, Spanish, and Portuguese were included. Studies with overlapping syndromes or genetic conditions were excluded.

**Data synthesis:**

After screening, 64 articles were included. A total of 205 individuals with Williams-Beuren syndrome diagnosed by FISH were included and further analyzed. Cardiovascular malformations were the most frequent finding (85.4%). Supravalvular aortic stenosis (62.4%) and pulmonary stenosis (30.7%) were the main cardiac alterations described.

**Conclusions:**

Our literature review reinforces that cardiac features may be the key to early diagnosis in Williams-Beuren syndrome patients. In addition, FISH may be the best diagnostic tool for developing nations that have limited access to new technologic resources.

## RESUMO

**Objetivo:**

Caracterizar todos os casos de síndrome de Williams-Beuren (SWB) diagnosticados por hibridização *in situ* fluorescente (FISH) desde sua implementação, assim como discutir a relação custo-benefício da metodologia de FISH em países em desenvolvimento.

**Fontes de dados:**

Entre janeiro de 1986 e janeiro de 2022 foi realizada uma busca nas bases de dados PubMed (Medical Literature Analysis and Retrieval System Online — Medline) e Scientific Electronic Library Online (SciELO) usando os seguintes termos: síndrome de Williams e hibridização *in situ* fluorescente. O critério de inclusão utilizado foi conter a descrição detalhada de caso(s) de SWB por FISH. Apenas estudos escritos em inglês, espanhol e português foram incluídos. Trabalhos que apresentavam sobreposição de síndromes/condições genéticas foram excluídos.

**Síntese dos dados:**

Após os processos de inclusão, 64 artigos e 205 indivíduos com SWB diagnosticados por meio do método de FISH foram incluídos. O achado mais frequente entre os indivíduos foi a presença de algum tipo de malformação cardíaca (85,4%). A estenose aórtica supravalvar (62,4%) e a estenose pulmonar (30,7%) foram as alterações cardíacas mais descritas. A maioria dos estudos era proveniente dos continentes Europa, Ásia e América do Norte.

**Conclusões:**

A presente revisão de literatura reitera que as malformações cardíacas podem ser a chave para o diagnóstico precoce em pacientes com SWB. Ainda, a técnica de FISH parece ser a melhor ferramenta de diagnóstico para os países em desenvolvimento, cujo acesso às novas tecnologias ainda é escasso.

## INTRODUCTION

Williams-Beuren syndrome (WBS) (OMIM #194050) is a rare developmental disorder with an autosomal dominant trait and numerous clinical findings. WBS is considered a contiguous gene syndrome caused by a microdeletion on chromosome 7q11.23 that ranges in size from 1.5 to 1.8 Mb and encompasses approximately 28 genes.^1,2^ The prevalence of the syndrome is estimated to be 1 per 7,500 live births.^3^ Distinctive facial features (long philtrum, epicanthal folds, broad forehead, bitemporal narrowness, periorbital fullness, stellate and/or lacy iris pattern, short nose with a bulbous nasal tip, wide mouth, full lips, and mild micrognathia), developmental and intellectual delay, an overly sociable personality, cardiovascular diseases, and idiopathic hypercalcemia comprise the overall WBS phenotype.^4,5^

Fluorescence in situ hybridization (FISH) analysis is considered the gold standard test for precise molecular diagnosis of microdeletion syndromes, such as WBS. Although new technologies such as array-CGH and multiplex ligation-dependent probe amplification (MLPA) outdated FISH, mostly in diagnosing atypical deletion cases, FISH is still a valuable and cost-effective tool to confirm WBS clinical suspicion.^2,6,7^

The area of rare diseases faces a lot of major obstacles with regard to gaining a deep understanding of each syndrome in order to improve patient care. In developing countries, some of these hurdles include difficulty in obtaining a timely and precise diagnosis, shortage of specialized healthcare workers, lack of research, and resource constraint.^8^ In Brazil, for example, the Universal Health Service, a large and well-established public healthcare system, does not offer molecular and genetic tests on a daily basis, which directly affects the diagnosis and management of patients with genetic rare diseases.^9^ Therefore, an ultimate molecular diagnosis of patients with rare genetic diseases is extremely important. A proper and precise diagnosis aids in the access of proper resources, avoids additional molecular investigation, decreases prognostic uncertainty, allows genetic counseling, and provides psychosocial benefits to both the patient and the family.^10^

The aim of this literature review was to sum up and characterize all WBS cases diagnosed by FISH since its implementation as well as to discuss FISH as a cost-effective methodology in developing countries.

## METHOD

This literature review was designed in accordance with the Preferred Reporting Items for Systematic Review and Meta-Analyses guidelines.^11^ The literature search was conducted using PubMed (Medline) and SciELO. Mesh and DECS descriptors were used to index articles with the following terms: Williams syndrome and In Situ Hybridization, Fluorescence. The exact search terms for PubMed/MESH terms were “Syndrome, Williams OR Contiguous Gene Syndrome, Williams OR Supravalvar Aortic Stenosis Syndrome OR Williams-Beuren Syndrome OR Syndrome, Williams-Beuren OR Williams Beuren Syndrome OR Beuren Syndrome OR Syndrome, Beuren OR Hypercalcemia-Supravalvar Aortic Stenosis OR Aortic Stenoses, Hypercalcemia-Supravalvar OR Aortic Stenosis, Hypercalcemia-Supravalvar OR Hypercalcemia Supravalvar Aortic Stenosis OR Hypercalcemia-Supravalvar Aortic Stenoses OR Stenoses, Hypercalcemia-Supravalvar Aortic OR Stenosis, Hypercalcemia-Supravalvar Aortic OR Chromosome 7q11.23 Deletion Syndrome OR Williams Contiguous Gene Syndrome) AND (FISH OR Hybridization in Situ, Fluorescent OR FISH Technique OR FISH Techniques OR Technique, FISH OR Techniques, FISH OR Fluorescent in Situ Hybridization OR FISH Technic OR FISH Technics OR Technic, FISH OR Technics, FISH OR Hybridization in Situ, Fluorescence OR In Situ Hybridization, Fluorescent,” and for SciELO/DECS, terms were “Williams Syndrome” AND “In Situ Hybridization, Fluorescence.”

Included articles were selected in a two-step analysis: title and abstract screening, followed by a full-text read. Authors were categorized into two pairs for independent screening and further discussion of potential disagreements (A.S./B.C. and D.D/P.S.). If the disagreement remained, a “senior reviewer” (B.D.) decided if the study would be included or excluded. Inclusion criteria for the first step were as follows: have a case or cases of WBS as well as any indication of FISH performance. In the second step, the inclusion criteria included WBS cases diagnosed by FISH with a stratified phenotype for each patient. Only studies from January 1986 to January 2022 written in English, Spanish, and Portuguese were included. Studies with overlapping syndromes or genetic conditions were excluded.

Publication metadata were extracted using a data extraction template that was created and modified according to all the studies reviewed. The publication details were captured and summarized in a tabular format developed by the authors of this review. The data extracted from all articles were as follows: article ID, total of cases, case stratification, auditory, behavioral, calcium, cardiovascular, cognitive, connective tissue, dental, endocrine, facial features, gastrointestinal, genitourinary, growth, hematology, integument, musculoskeletal, neurologic, ocular and visual, respiratory, tumor, “typical face,” sample type, gender, age at diagnosis with FISH, diagnosis with other molecular techniques beyond FISH, FISH probes, and authors’ countries.

## RESULTS

**Figure 1.**
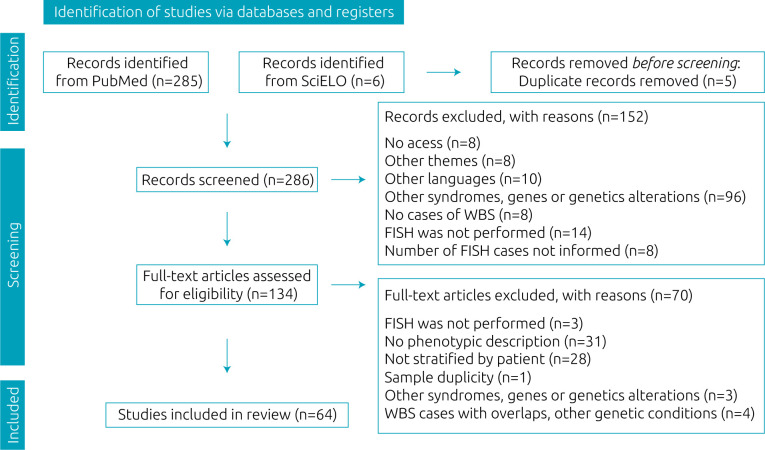
Flow diagram.

FISH: fluorescent in situ hybridization; WBS: Williams-Beuren syndrome.

After screening, 64 articles were included. A flow diagram of the literature search is depicted in Figure 1. A total of 205 individuals diagnosed with WBS by FISH were included and further analyzed. Demographic analysis showed that 48.5% (66/136) were female and 51.5% (70/136) male. Age at diagnosis ranged from 3 weeks to 37.75 years, with an average of 9.4 years and a median of 6.4 years. Clinical features were evaluated using the Guidance for Clinician in Rendering Pediatric Care.^5^ Cardiovascular malformations were the most frequent finding (85.4%), followed by neurological alterations (59.1%), cognitive delay (49.8%), facial dysmorphisms (48.3%), and behavioral changes (46.3%). Clinical findings are described in Table 1. A significant percentage of the patients (40.5%) did not have a clear description of their facial dysmorphisms, terms such as “typical face” or “elfin face” were used instead.

**Table 1.** Patients’ clinical findings by systems.

Main clinical features	Frequency (%)
Cardiovascular	85.4
Neurologic	59.1
Cognitive	49.8
Facial features	48.3
Behavioral	46.3
Growth	35.6
Connective tissue	29.8
Gastrointestinal	26.3
Integument	24.9
Dental	23.9
Auditory	20.0
Ocular and visual	18.5
Calcium	18.1
Musculoskeletal	13.7
Genitourinary	13.2
Respiratory	7.8
Endocrine	2.9
Hematology	2.9
Tumor	2.9

### Fluorescence in situ hybridization

Among the included studies, 53.1% did not report the probe(s) used for WBS diagnosis. The described probes are shown in Table 2.^12,13^ FISH analysis with more than one probe was performed in seven articles.^14,15,16,17,18,19,20^ In these studies, a variation in the deletion length was observed among patients since different probe sets were used. Other molecular and cytogenetics technologies were used in order to aid in the diagnosis of WBS. Microarray (9.3%) and microsatellite (6.3%) analysis were the most frequent techniques performed alongside FISH. MLPA, PCR, QMPSF, qPCR, and Southern analysis were also performed, and each of them comprised 4.4% of the included studies.

**Table 2.** FISH probes, BACs, cosmids, PACs, and YACs described and used by all included articles.

FISH probes	Genes	Diagnosed cases (n)
Commercial FISH probes		
WSCR probe (ONCOR, Gaithersburg, MD)	*ELN*	92
Q Biogene, currently MP Biomedicals, probe number CP5155-DC		1
Vysis LSI ELN Kit (Vysis; Abbott Laboratories, Abbott Park, IL)	*ELN+LIMK1*	29
MD Williams-Beuren Kreatech probe	*ELN+LIMK1+CYLN2*	1
Cytocell Williams-Beuren region probe	*LIMK1+EIF4H+RFC2+CYLN2+GTF2IRD1+TBL2+BAZ1B*	3
BACs/Cosmids/PACs/YACs		
cELN-272 and cELN-11D	*ELN*	9
Elastin cosmid		1
Cosmid P5155		1
*ELN* cosmid 82C and the *ELN/ LIMK1* cosmid 34B FISH probes	*ELN+LIMK1*	1
Cosmid probes	*ELN+LIMK1+STX1A*	4
CTB-8H17	FKBP6+FZD9+WSTF*	1
BACs 1008H17, 592D8, P195H06, 1148G03, 054H15; cosmids 182B11, 183E1	*FKBP6+FZD9+ELN+STX1A+GTF2IRD1+CYLN2+GTF2I*	1
Probes B315H11 and CITB51J22	*FZD9+BAZ1B+TBL2+LIMK1+RFC2* ^*†*^	1
BACs 1008H17, 315H11, 592D8, 155B1, 363B4; cosmids 12915, 82c2, 34b3, 152a8, 128d2, 102f12, 135f3, 82b11, 209c11, 47d1, 160g4, 183e1; PACs 632N4, 391G2, 195H6	*ELN+LIMK1+FZD9+FKBP6+BAZ1B+BCL7B+TBL2+ WBSCR14+STX1A+CLDN3+EIF4H+HSPCO46+RFC2+CYLN2*	3

*Genes referenced according to Korenberg et al.^12^; ^†^Genes referenced according to van Hagen et al.^13^. FISH: fluorescent in situ hybridization; WBS: Williams-Beuren syndrome; BAC: bacterial artificial chromosomes; PAC: P1-derived artificial chromosomes; YAC: yeast artificial chromosomes.

### Where, in theworld, are fluorescence in situ hybridization and Williams-Beuren syndrome studies from?

An overview of the authors’ locations is described by continents in Table 3. Europe, Asia, and North America comprised the highest percentage of studies included in this review. On the contrary, Latin America and Africa had the lowest numbers, with 4.7 and 3.1% of the studies, respectively. Among the countries, the United States (14.1%), Italy (12.5%), Japan (9.4%), and the United Kingdom (7.8%) were highlighted.

**Table 3.** Overview of the authors’ locations by continents.

Continents	Frequency (%)
Africa/Europe	3.1
Asia	26.6
Asia/North America	1.6
Eurasia	3.1
Europe	37.5
Europe/Eurasia	1.6
Europe/North America	6.3
Latin America	4.7
North America	15.6

## DISCUSSION

Initially, WBS diagnoses were made purely based on the clinical features observed in the patients. The observed phenotype should meet the descriptions provided by Williams et al.^21^ and Beuren et al.^22^ WBS was primarily described as a syndrome characterized by supravalvular aortic stenosis (SVAS), intellectual disability, facial dysmorphism, dental anomalies, and peripheral pulmonary artery stenosis. Further studies described additional dysmorphic features but without a proper etiology explanation.^23–25^ A WBS score (diagnostic index) was developed by Preus^26^ in order to assess syndrome features and provide patients’ diagnoses.

The term “elfin facies” was described in the 1970s to characterize all recurrent facial dysmorphisms found in WBS individuals.^24,27^ WBS facial phenotype is distinguished by a broad forehead, medial eyebrow flare, periorbital fullness, strabismus, stellate iris pattern, flat nasal bridge, malar flattening, full cheeks and lips, a long and smooth philtrum, a rather pointed chin, and a wide mouth.^28^ In our review, 40.5% of the included patients were described as having an “elfin face” or a “typical face.” The choice of a general description instead of detailed information regarding facial dysmorphisms in WBS patients may hinder a genuine clinical diagnosis of the syndrome. Since 1986, the use of generic terms to report WBS facial features has been discouraged.^28^ In our review, studies that provided a detailed description of patients’ dysmorphisms showed that the most prevalent features were full lips and a long philtrum (28.3%), followed by periorbital fullness (27.3%), wide mouth (26.3%), full cheeks (25.4%), and broad nasal tip (22.9%). A WBS patient’s clinical evaluation is extremely relevant in order to provide a clear and precise diagnosis. Heterogeneity between patients’ facial dysmorphism is also broadly described in the literature.^2^ The term “elfin face,” whose definition is based on a mythological and abstract figure, does not reflect the variety of facial features already described throughout WBS individuals. Therefore, the use of generic terms as part of the syndrome spectrum should be discouraged. Hence, we strongly recommend the use of standardized nomenclature to describe the facial phenotype of WBS patients.

Cardiovascular alterations (80%) are the most frequent features observed in WBS children and are also the major causes of infant morbidity and mortality within the syndrome.^5^ SVAS (75%) is considered the main cardiac finding observed in WBS patients, followed by pulmonary artery stenosis (50%).^5,29^ In our review, SVAS (62.4%) and pulmonary stenosis (30.7%) were the main cardiac alterations described. The high percentage of cardiovascular malformation among WBS patients points out the value of a detailed cardiovascular screening in an early clinical diagnosis of the syndrome. Although heart features are already known and often described within the syndrome spectrum, WBS patients’ diagnoses are still delayed (>1 year, on average).^29^ WBS neonatal diagnosis is challenging since some classical features include a friendly personality and facial dysmorphisms that are usually observed only days or months after birth. WBS clinical phenotype is also heterogeneous, and features tend to develop over time, which hinders a proper early clinical diagnosis.^30,31,32^ However, the main congenital heart diseases (CHDs) described in WBS can be screened and diagnosed through routine ultrasonography during the first trimester of pregnancy when performed by expert ultrasonographists.^33^ Therefore, we suggest that patients suspicious of WBS should go through a careful examination when looking for cardiovascular findings. As opposed to facial dysmorphisms that are observed over time, congenital heart diseases can be diagnosed early with the aid of prenatal ultrasound. Hence, in order to provide an early diagnosis for WBS patients, CHDs may be the golden key.

**Figure 2.**
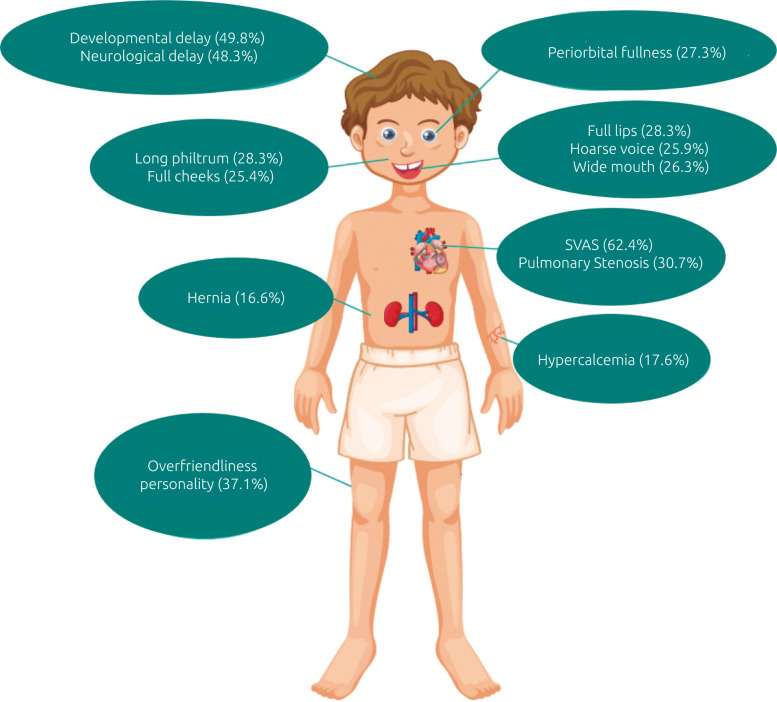
Representation of a male WBS patient with classical phenotypic features highlighted.

WBS: Williams-Beuren syndrome; SVAS: supravalvular aortic stenosis.

Developmental delay (90%) is often observed in WBS individuals. Therefore, referral to early intervention programs such as special education and vocational training is crucial to improve physical, speech, and nutrition features as well as social integration among patients.^4,5^ Intellectual delay ranging from light to moderate (75%) and a unique cognitive and behavioral profile are other features frequently described in WBS individuals.^5,34,35^ WBS children present an over-friendliness personality characterized by an intense drive for social interaction, a desire to form affectionate bonds, and an increased feeling of empathy. Therefore, an early intervention that aims to enhance social interactions and improve social skills is needed to ease the social inclusion of teenagers and adults with WBS.^35^ In our review, developmental delay was found in 49.8% of the patients. Intellectual delay and an over-friendliness personality were described in 48.3 and 37.1% of the individuals, respectively. An overall view of all phenotypic features is shown in Table 1 and Figure 2.

FISH was first performed by Pinkel et al.^36^ and Pinkel et al.^37^ This cytogenetic technique provides a rapid, precise, and reliable molecular analysis to confirm the suspicion of a clinical diagnosis. FISH is considered the gold standard method for chromosome microdeletion syndromes diagnosis.^4,6,38,39^ The WBS diagnosis rate of FISH is over 90% of the cases.^39^ WBS molecular etiology was described in the 1990s, the same decade that FISH was implemented as a diagnostic tool for WBS individuals.^40,41,42^ In 1993, Ewart et al.^1^ found that the molecular cause of WBS was a microdeletion at chromosome 7q11.23 after observing a elastin gene (*ELN*) hemizygosity by FISH. In our review, peripheral blood was the most commonly collected sample. Surprisingly, we found a case of postmortem diagnosis performed by FISH using both formalin-fixed tissues and paraffin-embedded sections from the kidney. Literature shows that buccal swabs can also be used as samples for FISH microdeletion analysis.^43^ Therefore, FISH proved to be an extremely versatile technique when it comes to sample types that can be used as a DNA source.

*ELN*, *LIMK1*, and *CYLN2* were the main genes designed within FISH probes used in the included studies. *ELN* encodes a structural protein that composes a diversity of body tissues and is the major gene associated with WBS. Therefore, *ELN* deletion can cause some connective tissue abnormalities such as cardiovascular diseases, in particular SVAS in WBS individuals.^1,4,44^ *LIMK1* deletion is associated with constructive visuospatial cognition abnormalities as well as neurological features in WBS patients.^45–47^ On the contrary, *CYLN2* is a gene associated with cerebellar malformation and neurological impairment that can lead to hippocampal dysfunction and a delay in the development of motor skills.^46,48^ Some studies performed other molecular methodologies alongside FISH that allowed a large number of genes to be identified as deleted as well as involved in WBS phenotype. However, the majority of these studies were conducted in developed countries. Therefore, FISH may be the better choice for developing nations that are still lacking in new technologies.

**Figure 3.**
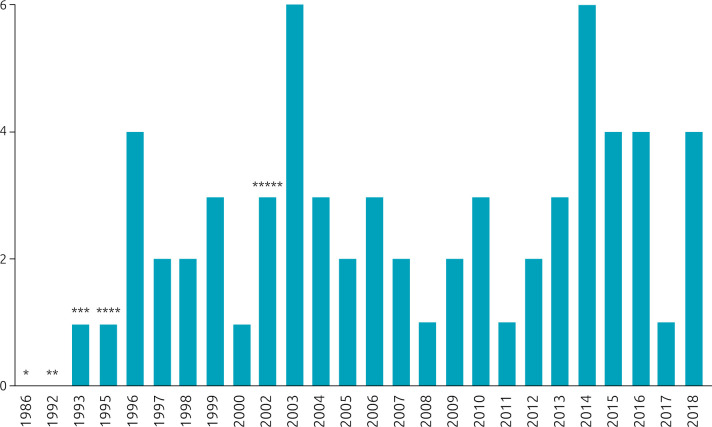
Included studies by year of publication. * FISH’s performed for the first time by Pinkel et al.^36,37^; ** CGH’s performed for the first time by Kallioniemi et al.^49^; *** Ewart et al.^1^ showed that WBS is caused by a microdeletion at chromosome 7q11.23 through FISH; **** a novel microsatellite DNA marker for WBS patients^51^; ***** MLPA is performed for the first time by Schouten et al.^52^. FISH: fluorescent in situ hybridization; WBS: Williams-Beuren syndrome; MLPA: multiplex ligation-dependent probe amplification; CGH: comparative genomic hybridization.

Comparative genomic hybridization (CGH) was first performed by Kallioniemi et al.^49^ in order to analyze solid tumor cytogenetics. Nowadays, this technology is widely used to detect chromosome copy number variation.^50^ The major advantage of genome-wide array platforms over FISH is the ability to screen for microdeletions and/or duplications throughout the genome that could detect not only WBS but also other syndromes at an earlier age.^50^ Gilbert-Dussardier et al.^51^ identified a novel microsatellite DNA marker (D7S1870) as a new diagnostic tool for hemizygosity detection in individuals suspected of WBS. Both CGH and microsatellites became valuable methodologies in the investigation of deletion length and characterization of microdeletion syndromes. In addition, MLPA was later added to the pool of molecular technologies that aid researchers in the diagnosis of WBS patientes.^52^ In this review, included studies dated from 1993 to 2018 (Figure 3)^1,36,37,49,51,52^. Although CGH, microsatellites, and MLPA were implemented in 1992, 1995, and 2002, respectively, FISH was still performed throughout the years.

### Fluorescence in situ hybridization X comparative genomic hybridization cost-effective analysis in developing countries

In the field of genetic rare diseases, molecular diagnosis is essential in order to elucidate the etiology of these conditions as well as provide a genotype-phenotype correlation for uncommon clinical outcomes.^10^ Nowadays, different and newer technologies are available to aid molecular investigation and further diagnosis. However, former standard methodologies, such as FISH, are still considered the gold standard for the detection of rare conditions, mainly in developing countries where financial support is limited and affordable technologies are preferred.^2,6,7,53–55^

FISH probes covering the *ELN* gene detect the majority of the deletion in children clinically diagnosed with WBS.^39,53,54^ Nickerson et al.^39^ showed that more than 90% of the patients were hemizygous for the elastin gene, while Souza et al.^53^ verified that 83% of the children clinically diagnosed with WBS had the same deletion. Moreover, Ramírez-Velazco et al.^54^ analyzed patients clinically diagnosed with WBS and identified that 66% of them had the 7q11.23 deletion detected by FISH. The study also performed CGH analysis in 23 cases where all FISH results were confirmed (18 deletions and 5 negatives). The additional information provided by CGH was the deletion sizes, which enables the patient’s classification into typical and atypical deletions.^54^ Both studies were conducted in developing countries (Brazil and Mexico), and the conclusion that FISH is the most feasible, effective, and economical approach in those nations was unanimous.^53,54^

In addition, a Brazilian study estimated the techniques’ budget in the United States and Brazil and revealed that, on average, US$600 is needed to perform both FISH and CGH analysis in the United States, while in Brazil, FISH analysis would cost US$800 and CGH analysis US$1200.^56^ Additionally, CONITEC, a committee that advises the Brazilian Ministry of Health, estimated the financial impact of molecular technologies in the country and evaluated the cost of FISH and CGH analysis at R$400 and R$2.000, respectively.^57^ Therefore, since all newer technologies are expensive, mainly in countries without proper research support, their use is recommended when FISH is negative in order to investigate atypical deletions.^55^ The lack of medical genetic services, health facility limitations, and healthcare access restrictions are also hardships faced by developing nations.^58^ Consequently, a proper evaluation by the government is needed in order to seek better healthcare services as well as improved research outcomes, mainly for patients with rare diseases such as WBS.^8^

Although our results are significant, some limitations of this review would be the restriction to access some potential studies that could contribute to our data. To include more articles published around the world, the inclusion of additional databases, such as Embase and Biblioteca Virtual em Saúde, would be interesting.

It is known that the clinical diagnosis alone is insufficient in order to give a proper treatment and follow-up for WBS patients. WBS has a heterogeneity of features described, and a lot of these characteristics are only observed after months or years of life. Therefore, the combination of a successful diagnostic rate for WBS individuals by FISH and a proper cardiac screening (mainly SVAS) may be the key for an early and precise diagnosis in neonatal patients with WBS. An early diagnosis is crucial since these individuals need a multiprofessional healthcare team in order to lessen and soften further complications as well as prevent secondary complications.^4,5^ In addition, FISH is a atemporal technology that seems to be irreplaceable for WBS diagnosis, mainly in countries that lack newer methodologies.
